# An mHealth Intervention Promoting Physical Activity to Reduce Psychological Distress Among Workers: Randomized Controlled Trial

**DOI:** 10.2196/96072

**Published:** 2026-08-14

**Authors:** Kazuhiro Watanabe, Akiomi Inoue, Asuka Sakuraya, Kotaro Imamura, Toru Yoshikawa, Akizumi Tsutsumi

**Affiliations:** 1 Department of Public Health, Kitasato University School of Medicine, 1-15-1 Kitazato, Minami-ku, Sagamihara, Kanagawa, 252-0374, Japan, 81 42-778-9352; 2 Department of Digital Mental Health, Graduate School of Medicine, The University of Tokyo, Tokyo, Japan; 3 Institutional Research Center, University of Occupational and Environmental Health, Japan, Kitakyushu, Fukuoka, Japan; 4 Research Center for Overwork-Related Disorders (RECORDs), National Institute of Occupational Safety and Health, Japan, Kawasaki, Kanagawa, Japan

**Keywords:** eHealth, behavioral change, smartphone, workplace, depression, anxiety

## Abstract

**Background:**

Although mobile health (mHealth) interventions serve as potential solutions for addressing mental health problems, evidence on whether mHealth interventions targeting physical activity can reduce psychological distress among generally healthy workers is limited.

**Objective:**

This study aimed to investigate the effectiveness of a stand-alone smartphone app, which passively monitors physical activity and psychological distress, in reducing psychological distress among workers.

**Methods:**

This open-label, 2-arm, parallel-group randomized clinical trial was conducted in Japan over 6 months, with assessments at baseline, 3 months, and 6 months. Eligible participants were workers aged 18 years or older who were able to complete the Japanese questionnaires and owned compatible smartphones. Individuals currently absent from work or with a history of absence due to sickness within the past 12 months were excluded. Participants were randomized (1:1) to the intervention or control group. Participants in the intervention group used a smartphone app that passively monitored physical activity and mental health for 3 months. The control group received a booklet on job stress. The primary outcome was psychological distress measured using the 6-item Kessler Psychological Distress Scale. Secondary outcomes included self-reported physical activity and digitally recorded activity duration in the intervention group. Analyses followed the intention-to-treat principle. For the main analysis, linear mixed modeling for repeated measures was used to estimate between-group differences in changes in psychological distress across the 3 time points.

**Results:**

A total of 793 workers were randomized (intervention: n=397; control: n=396). Psychological distress decreased significantly in the intervention group at 3 months (mean difference −0.56, 95% CI −1.11 to −0.02; *P*=.04; Cohen *d* −0.17), although this effect was not sustained at 6 months. Self-reported physical activity levels declined in both groups without significant between-group differences. In contrast, in the intervention group, digitally recorded activity increased during the intervention period (7.92 min increase, 95% CI 1.36-14.49; *P*=.02); however, these data were not comparable with those in the control group. Subgroup analysis showed a larger effect among participants with no or minimal baseline distress (mean difference −0.63, 95% CI −1.16 to −0.11; *P*=.02; Cohen *d* −0.19).

**Conclusions:**

The stand-alone smartphone-based app that enables workers to monitor physical activity and psychological distress modestly reduced psychological distress among workers. However, it remains unclear whether the app reduces psychological distress through biological and psychosocial mechanisms, including increased physical activity. Furthermore, the benefit may be short-lived rather than durable and may have limited clinical and practical significance. Although mHealth interventions may contribute to preventing mental health problems in workplace settings, booster engagement strategies are necessary to sustain benefits over time. Subgroup analyses suggested that the app may be more effective among generally healthy populations.

## Introduction

### Background

Many individuals in the working population experience mental health problems [1]. These range from common mental disorders such as depression and anxiety to subclinical symptoms, including psychological distress. Approximately 15% of working-age adults are estimated to have a mental disorder at any given time, and more than 250 million people worldwide live with depression or anxiety [2]. Additionally, several workers experience subclinical symptoms, including psychological distress [3,4]. These symptoms are consistently associated with increased sickness absence, reduced productivity, and lower quality of life and well-being [5]. Furthermore, surveys conducted by academic organizations indicate that over 70% of employees experience work-related stress [6]. Although workers are generally healthier than other populations, evidence-based strategies for the primary prevention of mental health problems remain a critical issue in occupational health.

Epidemiological evidence indicates that physical activity plays an important role in preventing and treating mental health problems. Physical activity interventions reduce symptoms of depression, anxiety, and psychological distress across diverse populations [7]. Both biological and psychosocial mechanisms through which physical activity improves mental health have been proposed, including neuroplasticity, neuroendocrine responses, inflammation, oxidative stress, self-esteem, self-efficacy, and social support [8,9]. Exercise appears comparable in effectiveness to psychological or pharmacological treatments for depression and may serve as an alternative or complementary approach [10]. Furthermore, the World Health Organization (WHO) recommends physical activity as an individual-level approach for promoting mental health in the workplace [2].

Mobile health (mHealth) interventions, particularly stand-alone smartphone apps, serve as potential solutions for addressing mental health problems [11]. By integrating smartphones into daily life, these tools provide accessible and personalized interventions. To encourage physical activity, mHealth interventions incorporate behavior change techniques, including feedback, self-monitoring, goal setting, rewards, and social comparison [12]. Evidence from various populations and settings supports their effectiveness in promoting physical activity [13-15]. Furthermore, several mHealth apps targeting physical activity have led to a reduction in depression, anxiety, and psychological distress [16,17].

Despite growing interest, evidence supporting mHealth physical activity interventions for improving mental health among the working population remains limited [18,19]. The most robust evidence comes from mHealth trials focusing on clinical or high-risk populations, such as individuals diagnosed with depression or with elevated baseline symptom levels. Moreover, physical activity interventions in workplace settings have primarily targeted physical health or physiological outcomes rather than mental health effects [20,21]. This research highlights a lack of rigorous randomized controlled trials (RCTs) examining whether mHealth interventions promoting physical activity can reduce psychological distress among generally healthy workers.

### Objectives

This study evaluated whether the stand-alone smartphone app ASHARE (Kitasato Institute) could reduce psychological distress among workers using an RCT design. ASHARE was developed to enhance workers’ mental health by integrating the monitoring of physical activity and psychological distress. The app incorporates a deep learning model that passively monitors psychological distress based on users’ physical activity patterns [22]. Earlier findings indicated that ASHARE was not more effective than an existing multicomponent workplace program, although reductions in psychological distress were observed in an ad hoc analysis of protocol-compliant participants [23,24].

The present trial rigorously investigated the individual-level effectiveness of ASHARE, targeting a broadly defined working population, including individuals with minimal or no psychological distress. We hypothesized that the intervention group would show greater reductions in psychological distress and greater increases in physical activity than the active control group.

## Methods

### Trial Design

This study was an open-label, 2-arm, parallel-group, RCT. After completing the baseline online survey, eligible workers were randomly assigned (1:1) to the ASHARE intervention or an active control condition that provided a booklet containing basic information about stress and simple individual exercises. Randomization was stratified by baseline psychological distress severity. Participants received their assigned program for 3 months, and follow-up surveys were conducted at 3 and 6 months to assess outcome changes. Psychological distress was the primary outcome. Subjective physical activity levels and digitally recorded activity durations were evaluated as secondary outcomes. Additionally, implementation outcomes—acceptability, appropriateness, feasibility, and satisfaction—as well as potential harm were assessed.

The intervention and control programs were originally planned within the M-ORION (Multifaceted Organizational Interventions) project, a 5-arm cluster RCT designed to evaluate 4 types of preventive interventions for depression and anxiety among workers [25]. Following a protocol revision to a simpler 2-arm design, the ASHARE intervention was implemented as an individual-level RCT. The protocol for the present trial was registered with the University Hospital Medical Information Network Clinical Trials Registry (ID UMIN000057908) on May 19, 2025 [26]. Recruitment of new participants ended on August 19, 2025. This report conformed to the CONSORT (Consolidated Standards of Reporting Trials) 2025 guidelines (Checklist 1) [27].

### Trial Setting

The RCT was conducted over 6 months, from late June 2025 to early January 2026. Participants were recruited through an internet survey company, Cross Marketing Inc [28], which was commissioned by the research team and used to identify potential participants across all prefectures in Japan through its respondent panel. For recruitment, the company distributed a web-based screening questionnaire to up to 10,000 individuals, including nonworkers, from its panel of over 14 million individuals in Japan. The questionnaire included items on eligibility criteria, trial information, and willingness to participate. The company then identified eligible individuals who expressed interest in participation and provided their information to the research team. Subsequently, the research team obtained informed consent and conducted a baseline survey to determine final eligibility.

### Ethical Considerations

The Kitasato University Medical Ethics Organization approved the study protocol (C24-179). Prior to screening and baseline assessments, participants received information about the study objectives, and informed consent was obtained. Participants were informed that the data would be managed on a secure cloud server at Kitasato University and would be deidentified for statistical analyses. A 500-yen (US $3.5 as of July 1, 2025) Amazon gift certificate was provided to participants as an incentive after completing each survey wave (baseline, 3-month follow-up, and 6-month follow-up). Compensation was provided equally to all participants, regardless of intervention adherence.

### Participants

The trial targeted workers in Japan. Eligibility criteria were (1) aged 18 years or older, (2) ability to complete questionnaires in Japanese, and (3) ownership of a personal smartphone compatible with the ASHARE app (Android version 5.0 or later, iOS version 12.0 or later). Participants were excluded if they (1) were absent from work at the time of enrollment or (2) had experienced work absence due to sickness during the 12 months prior to enrollment. Participants of all self-reported genders were eligible for inclusion (men, women, and those who preferred not to respond). Race and ethnicity data were not collected.

### Randomization and Masking

Eligible participants were randomized to the intervention or control group in a 1:1 ratio. To account for this important prognostic factor and to facilitate a subgroup analysis, randomization was stratified into 2 strata based on baseline psychological distress levels (minimal or no, 6-item Kessler Psychological Distress Scale [K6] score <5; subthreshold to severe, K6 score ≥5). Permuted block randomization with a block size of 4 was used to ensure a balanced allocation. An independent biostatistician at a private statistics company [29], who was blinded to the research team, generated the randomization sequence. A research assistant, who was blinded to the intervention providers, managed the allocation sequence and notified the principal investigator of the randomization results. As both groups received psychosocial interventions, allocation was not concealed from the participants, intervention providers, outcome assessors, or data analysts.

### Interventions

#### Intervention Group

Participants in the intervention group were asked to install the ASHARE app on their smartphones and use it for 3 months [30]. Details of the intervention content have been described in previous feasibility and nonrandomized trials [23,24]. ASHARE was developed to reduce psychological distress among workers by promoting physical activity. It incorporates basic behavior change techniques, including self-monitoring, feedback, rewards, and social comparison. The app enables users to monitor their 24-hour physical activity patterns and step counts through Google Health Connect (Android) and Apple Health (iOS). The duration of moderate-to-vigorous physical activity was calculated in 15-minute increments. Additionally, the app allowed the passive monitoring of psychological distress using an embedded deep learning model [22]. Based on a long short-term memory, the model estimated the next day’s psychological distress score from physical activity patterns and demographic characteristics (age group, gender, occupation, employment status, work shift type, and weekly working hours). A 5-fold cross-validation indicated that the overall classification accuracy of the deep learning model for psychological distress was 76.3%, with particularly high accuracy for minimal or no psychological distress. Since the model’s development, it has been periodically fine-tuned using users’ 3-level feedback (ie, estimation is correct, feeling better than estimated, and feeling worse than estimated). Moreover, rule-based feedback and data sharing among users were provided to enhance awareness of physical activity and psychological distress levels. A gamified leveling-up program was incorporated to promote user engagement, wherein users earned points each time they logged in. Furthermore, the app delivered daily smartphone notifications at 6 AM, prompting users to launch the app and monitor their psychological distress scores.

After random allocation, participants received a link via email from the principal investigator (KW) and installed the free ASHARE app from Google Play or the App Store. Participants were not required to use the app and could access it at any time during the intervention period. Participants were expected to spend approximately 5 minutes per day using the app to review their physical activity patterns and psychological distress and to modify their physical activity behaviors.

As part of the modifiable components designed to promote program adherence, the principal investigator conducted a 30-minute online information session via Zoom (Zoom Communications, Inc) in early July 2025. Participants had the option to attend any of the 7 sessions that provided identical content. The sessions were recorded and made available on YouTube during the intervention period. In addition, the principal investigator sent biweekly reminder emails encouraging participants to use the app. User log-in frequency was objectively monitored by the research team using the app’s administrative dashboard.

#### Active Control Group

Participants in the control group were instructed to download and read a booklet on job stress at any time during the 3-month study period. This program was designed as a basic, minimal intervention to provide participants with information about work-related stress. The details of the program have been described elsewhere [25]. The booklet was a 14-page PDF describing the job stress model, the association between stress and performance, and stressful life events. Additionally, it included a worksheet for participants to record acute reactions likely to occur in stressful situations. After random allocation, participants received a link via email from the principal investigator to download the booklet. No additional support beyond the distribution of the booklet was provided following the notification of the download link.

### Outcomes

#### Psychological Distress

The primary outcome was psychological distress, assessed at 3 time points (baseline, 3-month follow-up, and 6-month follow-up) using the K6 scale [31]. The K6 is a 6-item scale that assesses the frequency of symptoms related to depression and anxiety on a 5-point Likert scale from 0 (“none of the time”) to 4 (“all of the time”). The Japanese version has shown excellent criterion validity for mood and anxiety disorders [32]. In the present trial, the total K6 score was used in the primary analysis. Baseline K6 scores were further used for stratified randomization based on the cutoff for depression: minimal or no distress (<5) and subthreshold to severe distress (≥5). This optimal cutoff point yielded the highest Youden Index, with a sensitivity of 100% and moderate-to-high specificity for identifying mood or anxiety disorders [33].

#### Physical Activity

Secondary outcomes included subjective and objective measures of physical activity. The Japanese version of the Global Physical Activity Questionnaire (GPAQ) was used to assess self-reported physical activity levels at 3 time points [34]. The GPAQ is commonly used in epidemiological studies to assess physical activity levels and has reported acceptable reliability and convergent validity with established questionnaires in several countries, including Japan [35]. According to the GPAQ analysis guidelines, physical activity levels were classified into 3 ordinal categories: low, moderate, and high. Additionally, weekly physical activity (MET [metabolic equivalent of task]-min/wk) was calculated for occupational, transport, leisure-time activity, and total physical activity domains [36].

For the intervention group, digitally recorded activity duration (min/d) was evaluated as an objective indicator during the intervention period. This information was derived using proprietary algorithms embedded in Google Health Connect (Android) and Apple Health (iOS). These application programming interfaces recorded moderate-to-vigorous physical activity in 15-minute increments, and ASHARE transmitted this information to its cloud server. Physical activity measured using smartphones has demonstrated acceptable validity compared with ActiGraph measurements in free-living settings [37,38]. The values were aggregated on a per-day basis and used in the analyses.

#### Implementation and Harms

In the intervention group, user retention was calculated using log-in frequency data obtained from the app’s administrative dashboard during the intervention period. Retention was assessed weekly, and continuous users were defined as participants who used the app at least once per week. In the control group, adherence to the booklet (ie, whether participants downloaded and read it) was assessed at the 3-month follow-up.

Acceptability, appropriateness, feasibility, satisfaction, and potential harms of the intervention were assessed using the Implementation Outcome Scale of Digital Mental Health (iOSDMH) [39]. This 19-item instrument uses a 4-point Likert scale from 1 (“disagree”) to 4 (“agree”) and evaluates 5 domains related to the implementation and harms of digital mental health interventions. The scale has demonstrated known-groups and convergent validity in previous research [39]. In this study, an overall implementation score was calculated from the acceptability, appropriateness, feasibility, and satisfaction domains and included in the analyses. Potential harms were monitored using the harms domain score of the iOSDMH at the 3-month follow-up. A data monitoring committee, comprising the principal investigator and a senior supervisor (AT), oversaw safety monitoring. The trial was prespecified for termination if significant harm exceeded those reported in prior digital mental health studies [40].

### Sample Size Calculation

The required sample size was based on the effect sizes reported in previous studies and minimally important differences. A meta-analysis of mHealth interventions targeting physical activity reported standardized mean differences ranging from 0.18 to 0.42 [41]. Although evidence is limited for psychological distress, a distribution-based approach has suggested that a change of 0.2 SDs represents a small but minimally important change in patient-reported outcomes [42]. Accordingly, we specified the minimal detectable effect size at 0.2, with a 2-sided α of .05 and statistical power of 0.80. The required sample size was estimated as 394 participants per group (total N=788). Statistical power was calculated using G*Power version 3.1.9.2 (Heinrich Heine University Düsseldorf) [43].

### Statistical Analysis

To examine the effects of ASHARE on psychological distress, linear mixed modeling for repeated measures was used to estimate between-group differences in changes in K6 scores across the 3 time points. A mixed procedure was used in Stata version 19 (StataCorp LLC). The group × time interaction was represented as the primary indicator of the intervention effect. The level of psychological distress at baseline (0=“minimal or no distress” and 1=“subthreshold to severe distress”), which was used for stratified randomization, was included as a covariate. Based on model estimates, the adjusted means and SEs at each time point were obtained using the nonlinear combination (nlcom) command. Furthermore, effect sizes (Cohen *d*) and their SEs at each follow-up were calculated by dividing the estimated intervention effects by the pooled SD of the changes in K6 scores. The effectiveness of the intervention was assessed in subgroups based on the psychological distress level at baseline.

For the physical activity levels (low, moderate, and high) measured using the GPAQ, a generalized linear mixed model with a cumulative logit link was used to treat physical activity as an ordinal outcome. The model estimated the cumulative odds ratio (OR) for being in a higher physical activity category in the intervention group under the proportional odds assumption. The meologit command in Stata was used for this analysis. We further calculated the probability of each level in the 2 groups using the nlcom command. In the sensitivity analysis, weekly physical activity (MET-min/wk) was analyzed using a linear mixed effects model, as was done for psychological distress. Additionally, a subgroup analysis based on baseline levels of psychological distress was conducted.

These analyses were conducted based on the intention-to-treat principle, and all assigned participants were included in the analyses. As the online surveys required respondents to complete all items before submission, no item-level missing data occurred. However, missing data occurred due to attrition in the 3- and 6-month follow-up surveys. Missing values were addressed using multiple imputation. A total of 50 imputed datasets were generated based on the baseline information. Predictive mean matching was applied for continuous outcomes (psychological distress and amount of physical activity) and ordinal logistic regression was used for ordinal outcomes (physical activity level). The mi impute command was employed to generate the imputed datasets, and the mi estimate command was used to combine the results in Stata.

Among participants in the intervention group who installed and launched the ASHARE app, log-in frequency and digitally recorded activity duration were analyzed. The weekly mean activity duration (min/d) during the intervention period (wk 1‐13) was estimated using linear mixed modeling. Baseline psychological distress levels and the number of weeks for which the app was used were included as covariates. Furthermore, the interaction between time and the number of weeks of app use was examined.

As an ad hoc analysis, a per-protocol analysis was conducted to estimate the effect of the intended intervention. Adherent participants were defined as those who completed all follow-up surveys; in the intervention group, those who installed and used the app throughout the 13-week intervention period, and, in the control group, those who downloaded the booklet.

### Patient and Public Involvement

During app development, qualitative interviews were conducted with 12 participants to identify needs for mHealth services targeting physical activity and mental health [44]. For the trial design, a cluster RCT would have been ideal to evaluate the effectiveness of workplace interventions. However, a prior nonrandomized trial [24] and the originally planned trial [25], including qualitative input from health promotion managers and employees, indicated that randomization at the work unit or worksite level would not align with organizational decision-making processes and might reduce participation rates. Such challenges could increase the risk of selection bias, attrition, and deviation from the intended intervention. Therefore, an individual-level RCT was conducted to recruit workers who were motivated to participate.

### Deviations From the Protocol

Seven 30-minute online sessions via Zoom for the intervention group were not prespecified in the registered protocol; they were added as modifiable components to promote program adherence. Additionally, while the stratified randomization by baseline psychological distress level was prespecified, the corresponding subgroup analyses were not prespecified and were conducted post hoc.

## Results

### Study Flow and Participant Characteristics

**Figure 1.**
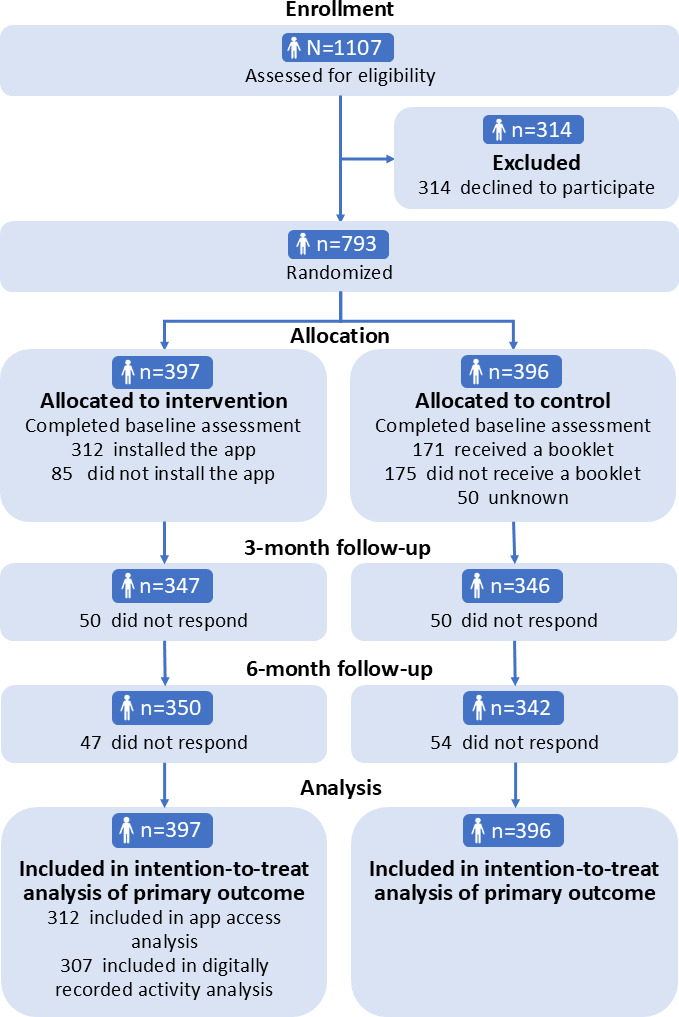
Participant flow diagram.

Figure 1 shows the participant flow diagram of the trial. The baseline survey and eligibility assessment were conducted over 4 days, from June 25, 2025, to June 28, 2025. Of the 1107 workers who expressed interest in participating in the trial, 793 met the eligibility criteria. The random allocation was performed by a research assistant and communicated to the research team on June 30, 2025. The principal investigator notified eligible participants of their allocation and program initiation on July 1, 2025. Following the 3-month intervention period, a 3-month follow-up survey was conducted from September 25, 2025, to October 14, 2025, and a 6-month follow-up survey was conducted from December 25, 2025, to January 15, 2026. The follow-up rates were 87.4% and 87.3% at 3 and 6 months, respectively.

Table 1 shows the baseline characteristics of the study participants. Among the 793 participants included in the study, 397 were assigned to the intervention group and 396 to the control group. More than half of the participants were men. The majority were regularly employed, worked day shifts, and held clerical positions. Overall, baseline characteristics were well balanced between the 2 groups, with no statistically significant differences. Subthreshold levels of severe psychological distress were present in 42.9% of participants in both groups.

**Table 1.** Baseline characteristics of the intention-to-treat population (N=793).

Characteristic	Intervention (n=397), n (%)	Control (n=396), n (%)
Age (y)		
20‐29	31 (7.8)	29 (7.3)
30‐39	87 (21.9)	124 (31.3)
40‐49	121 (30.5)	101 (25.5)
50‐59	93 (23.4)	84 (21.2)
≥60	65 (16.4)	58 (14.6)
Gender		
Men	208 (52.4)	219 (55.3)
Women	188 (47.4)	177 (44.7)
Not responded	1 (0.3)	0 (0)
Employment status		
Regular	250 (63.0)	268 (67.7)
Part time	83 (20.9)	62 (15.7)
Dispatched	20 (5.0)	17 (4.3)
Contract	21 (5.3)	25 (6.3)
Other	23 (5.8)	24 (6.1)
Shift type		
Day shift	347 (86.4)	351 (88.6)
Rotation shift	29 (7.3)	31 (7.8)
Night shift	8 (2.0)	1 (0.3)
Other	13 (3.3)	13 (3.3)
Occupation		
Manager	48 (12.1)	48 (12.1)
Professional/engineer/academic	85 (21.4)	85 (21.5)
Clerk	120 (30.2)	125 (31.6)
Sales	25 (6.3)	37 (9.3)
Services	59 (14.9)	37 (9.3)
Transportation	8 (2.0)	6 (1.5)
Construction	7 (1.8)	5 (1.3)
Production/skilled	15 (3.8)	24 (6.1)
Agriculture/forestry/fisheries	4 (1.0)	2 (0.5)
Other	26 (6.5)	27 (6.8)
Working hours (per week; hours)		
1‐40	208 (52.4)	193 (48.7)
41‐50	127 (32.0)	153 (38.6)
51‐60	39 (9.8)	27 (6.8)
61‐70	6 (1.5)	12 (3.0)
≥71	17 (4.3)	11 (2.8)
Level of psychological distress (K6)		
0‐4 (no or minimal)	227 (57.2)	226 (57.1)
5‐24 (subthreshold to severe)	170 (42.8)	170 (42.9)

### Program Delivery

**Figure 2.**
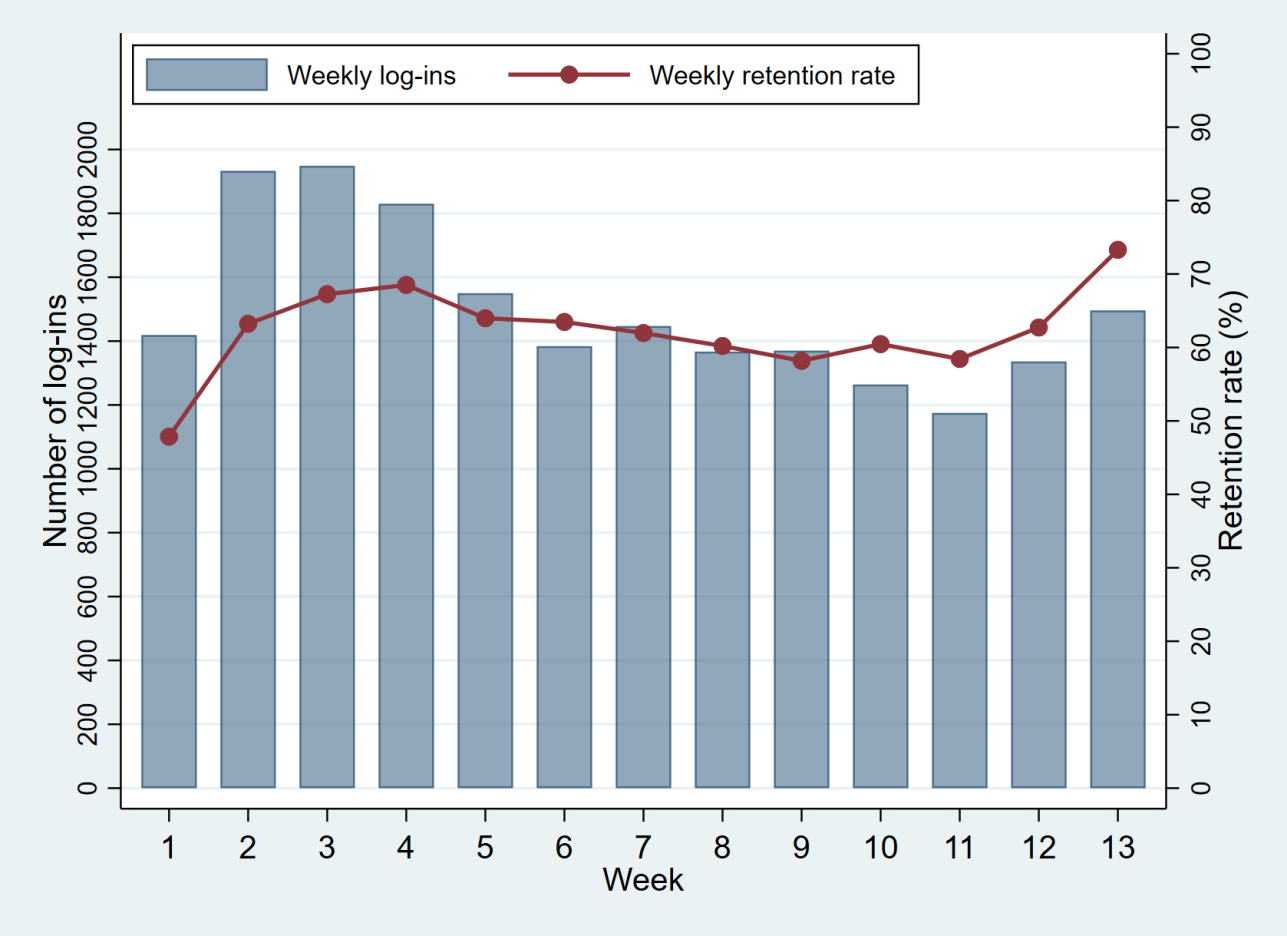
Number of log-ins and retention rate of the ASHARE app in the intervention group. The weekly number of log-ins was defined as the total number of app launches recorded during each week. The retention rate was calculated as the percentage of participants assigned to the intervention group who accessed the app at least once during a given week.

Following the program initiation email, seven 30-minute online information sessions were held for the intervention group on July 4, 5, 6, 10, 11, 12, and 13, 2025. Across the 7 sessions, 157 out of 397 (39.5%) attendances were recorded. Among participants in the intervention group, 312 (78.6%) installed ASHARE, and 307 linked their smartphone-based physical activity data. The average total number of log-ins across participants during the intervention period was 1501 (SD 239.6), peaking in the third week after baseline and corresponding to 3.8 log-ins per participant week (Figure 2). The mean retention rate was 62.3% (SD 5.8%), ranging from 47.9% to 73.3%, and participants used ASHARE for approximately 8 weeks. By contrast, at the 3-month follow-up, 43.1% (171/396) of the participants in the control group downloaded the booklet. The total number (N=793) of adherent participants was 279 (35.2%), including 120 in the intervention group and 159 in the control group. During the follow-up period (weeks 14‐26), the retention rate for the ASHARE app declined steeply, with a mean of 37.4% (SD 6.4%, ranging from 31.0% to 52.9%).

Implementation outcomes and the potential harm of ASHARE were evaluated at the 3-month follow-up (Multimedia Appendix 1). The total mean score of the iOSDMH (acceptability, appropriateness, feasibility, and satisfaction) was 39.74 (SD7.4), which was greater than those reported in the previous feasibility trial [23], non-RCT [24], and in half of prior digital mental health studies [39]. Among the subscales, the feasibility score was higher than those reported in the above benchmarks. The mean harm score was 7.36 (SD 3.0), which was the lowest among those reported in the above benchmarks. Few participants reported physical (31/347, 8.9%) or mental (27/347, 7.8%) symptoms, or safety-related dangerous experiences (20/347, 5.8%), associated with app use. However, 19.9% (69/347) reported feeling excessive pressure to use the app regularly. Another commonly reported harm was that app use consumed time (48/347, 13.8%).

### Effectiveness of the Intervention on Psychological Distress

Table 2 shows the estimated mean psychological distress scores at the 3 time points. The group × time interaction was significant at the 3-month follow-up, indicating that the reduction in psychological distress was greater in the intervention group (mean difference −0.56, 95% CI −1.11 to −0.02; *P*=.04). The corresponding effect size (Cohen *d*), calculated using the pooled SD of change scores, was −0.17 (95% CI −0.33 to −0.01). The between-group difference at the 6-month follow-up was small and not statistically significant (mean difference −0.15, 95% CI −0.69 to 0.39; *P*=.58).

**Table 2.** Effects of the intervention program on psychological distress (N=793)^a^.

Time points	Estimated mean (95% CI)		Differences in change scores of outcomes			
	Intervention (n=397)	Control (n=396)	Estimated mean difference (95% CI)	Pooled SD	Effect size (Cohen *d*, 95% CI)	*P* value
Baseline	5.01 (4.69 to 5.33)	5.04 (4.73 to 5.36)	—^b^	—	—	—
3-month follow-up	4.60 (4.16 to 5.05)	5.20 (4.73 to 5.68)	−0.56 (−1.11 to −0.02)	3.31	−0.17 (−0.33 to −0.01)	.04
6-month follow-up	4.99 (4.55 to 5.44)	5.18 (4.71 to 5.65)	−0.15 (−0.69 to 0.39)	3.30	−0.05 (−0.21 to 0.12)	.58

a The estimated mean scores were adjusted according to the level of psychological distress at baseline (0=no or minimal distress; 1=subthreshold to severe distress).

b Not available.

### Effectiveness of the Intervention on Physical Activity

Table 3 shows the estimated probabilities of physical activity levels and cumulative ORs for level changes. In Japan, from summer (July 2025, baseline) to winter (December 2025, 6-month follow-up), physical activity levels decreased in both groups, with no statistically significant between-group differences observed at either follow-up. The change in the physical activity level in the intervention group was unfavorable at the 3-month follow-up (cumulative OR 0.74, 95% CI 0.46-1.21; *P*=.24).

**Table 3.** Effects of the intervention program on the level of physical activity (GPAQ^a^; N=793)^b^.

Time points	Estimated probability (%) (95% CI)		Differences in change levels		
	Intervention (n=397)	Control (n=396)	Estimated difference (95% CI)	Cumulative OR^c^ (95% CI)	*P* value
Baseline			—^d^	—	—
High	12.4 (8.2 to 16.6)	11.4 (7.5 to 15.3)			
Moderate	64.9 (60.9 to 69.0)	64.2 (59.8 to 68.6)			
Low	22.7 (16.1 to 29.2)	24.4 (17.5 to 31.3)			
3-month follow-up			−0.29 (−0.77 to 0.19)	0.74 (0.46 to 1.21)	.24
High	10.0 (6.3 to 13.7)	11.9 (7.7 to 16.1)			
Moderate	62.8 (57.7 to 67.9)	64.6 (60.3 to 68.9)			
Low	27.2 (19.5 to 34.9)	23.5 (16.5 to 30.4)			
6-month follow-up			0.31 (−0.17 to 0.80)	1.37 (0.84 to 2.22)	.20
High	9.5 (6.0 to 12.9)	6.5 (3.9 to 9.1)			
Moderate	62.1 (56.8 to 67.5)	56.1 (49.1 to 63.2)			
Low	28.4 (20.6 to 36.2)	37.4 (28.4 to 46.4)			

a GPAQ: Global Physical Activity Questionnaire.

b High: ≥3 days of vigorous intensity activity for ≥1500 MET-min/wk or ≥7 days of any combination of walking or moderate-to-vigorous intensity physical activity for 3000 MET-min/wk; moderate: ≥3 days of vigorous intensity activity for ≥20 min/d or ≥5 days of moderate intensity activity or walking for ≥30 min/d or ≥5 days of any combination of walking or moderate-to-vigorous intensity physical activity for ≥600 MET-min/wk; low: not meeting the criteria for high or moderate. The odds ratios were adjusted according to the level of psychological distress at baseline (0=no or minimal distress; 1=subthreshold to severe distress).

c OR: odds ratio.

d Not available.

When physical activity was analyzed as weekly volume (MET-min/wk), the total physical activity did not increase in either group but remained stable in the intervention group at the 3-month (mean difference 208.03, 95% CI −392.31 to 808.37; *P*=.50) and 6-month (mean difference 327.10, 95% CI −265.94 to 920.15; *P*=.28) follow-ups; however, these differences were not statistically significant (Multimedia Appendix 2). Among the activity domains, the largest difference in change was observed in transport-related activity.

The weekly mean activity duration based on digitally recorded ASHARE data showed a different trend. Among the 307 participants in the intervention group, the mean daily physical activity duration increased by 7.92 minutes during the intervention period (95% CI 1.36-14.49; *P*=.02), rising from week 1 (57.88 min/d, 95% CI 48.07-67.70) to week 13 (65.81 min/d, 95% CI 57.55-74.06). Moreover, the interaction with the number of weeks of app use was positive (coefficient 0.18, 95% CI 0.01-0.36; *P*=.04), indicating that participants who used ASHARE for longer periods were more likely to increase their activity duration (Multimedia Appendix 3).

### Subgroup Analysis Based on the Level of Psychological Distress at Baseline

Tables 4 and 5 show the results of the subgroup analysis stratified by baseline psychological distress levels. Overall, outcome patterns were similar to those observed in the primary analysis. However, the effect on psychological distress was significant and larger among participants with no or minimal distress (mean difference −0.63, 95% CI −1.16 to −0.11; *P*=.02; Cohen *d* −0.19) than among those with subthreshold to severe distress. Estimated mean trajectories demonstrated that increases in psychological distress were attenuated in the intervention group.

**Table 4.** Subgroup analysis: effects of the intervention program on psychological distress (K6) by levels of psychological distress.

Time points	Differences in change scores of outcomes					
	No or minimal (K6<5) (n=453)			Subthreshold to severe (K6≥5) (n=340)		
	Estimated mean difference (95% CI)	Effect size (Cohen *d*) (95% CI)	*P* value	Estimated mean difference (95% CI)	Effect size (Cohen *d*) (95% CI)	*P* value
3-month follow-up	−0.63 (−1.16 to −0.11)	−0.19 (−0.35 to −0.03)	.02	−0.47 (−1.48 to 0.53)	−0.14 (−0.45 to 0.16)	.36
6-month follow-up	−0.31 (−0.82 to 0.20)	−0.09 (−0.25 to 0.06)	.23	0.05 (−0.96 to 1.06)	0.02 (−0.29 to 0.32)	.92

**Table 5.** Subgroup analysis: effects of the intervention program on physical activity by levels of psychological distress (GPAQ^a^).

Time points	Differences in change levels of outcomes					
	No or minimal (K6<5) (n=453)			Subthreshold to severe (K6 ≥5) (n=340)		
	Estimated difference (95% CI)	Cumulative OR^b^ (95% CI)	*P* value	Estimated difference (95% CI)	Cumulative OR (95% CI)	*P* value
3-month follow-up	−0.36 (−1.00 to 0.27)	0.70 (0.37 to 1.31)	.26	−0.20 (−0.95 to 0.55)	0.82 (0.39 to 1.73)	.60
6-month follow-up	0.41 (−0.22 to 1.05)	1.51 (0.81 to 2.84)	.20	0.17 (−0.57 to 0.91)	1.18 (0.56 to 2.48)	.66

a GPAQ: Global Physical Activity Questionnaire.

b OR: odds ratio.

When physical activity was analyzed as weekly volume (MET-min/wk), increases in total and transport-related activity were statistically significant among participants with subthreshold to severe distress; however, the SEs of the estimates were large (Multimedia Appendix 4).

### Per-Protocol Analysis Among Adherent Participants

The per-protocol analysis (Multimedia Appendix 5) among adherent participants (n=279) indicated a greater reduction in psychological distress at the 3-month follow-up (mean difference −0.92, 95% CI −1.65 to −0.18; *P*=.01; Cohen *d* −0.28) and at the 6-month follow-up (mean difference −0.50, 95% CI −1.28 to 0.28; *P*=.21; Cohen *d* −0.15). For physical activity, although levels decreased in both groups, the decline was more gradual in the intervention group at both follow-ups. When physical activity was analyzed as weekly volume (MET-min/wk), a greater increase in leisure-time physical activity was observed in the intervention group at the 3-month follow-up (mean difference 289.40, 95% CI 17.20-561.60; *P*=.04).

## Discussion

### Principal Findings

The findings revealed that the ASHARE app produced a modest but statistically significant reduction in psychological distress in the immediate postintervention assessment. To our knowledge, this is the first RCT to demonstrate the effectiveness of a smartphone-based intervention centered on a stand-alone app, with additional engagement supports, that integrates the monitoring of both physical activity and psychological distress in a working population, including individuals with minimal or no psychological distress. However, this effect was not sustained at the 6-month follow-up; the benefit may be short-lived and not durable. Furthermore, the estimated effect size (−0.17) was smaller than the expected minimally important change (−0.20). The proportion of participants reporting subthreshold to severe psychological distress (K6≥5) decreased by only 3.7% in the intervention group. Therefore, the observed effect may have limited clinical and practical significance. Although mHealth interventions may contribute to the primary prevention of mental health problems in the workplace, booster engagement strategies are necessary to sustain benefits over time.

The magnitude of the effect size for psychological distress was small, which was consistent with expectations for digital interventions conducted among generally healthy workers [45,46]. Moreover, the short-term nature of the intervention effect has been repeatedly observed in previous studies [46,47]. By contrast, interventions that directly deliver structured physical activity or exercise sessions tend to report larger effect sizes [7,48], and these effects are often maintained over longer periods (eg, over 6 months) [13]. These differences may highlight the nonintensive, self-guided nature of the present intervention in the working population. This interpretation is supported by the steep decline in retention rates during the follow-up period (weeks 14‐26). To sustain benefits and prevent the worsening of psychological distress, booster engagement strategies for app use, such as additional educational sessions and reminders, may be required.

Physical activity levels declined in both groups; it remains unclear whether the ASHARE app reduces psychological distress through increased physical activity. The overall decline may partly reflect seasonal variation, as the follow-up period extended from summer to winter, when activity levels typically decrease [49]. In contrast, mixed results were observed in the domain-stratified, subgroup, and per-protocol analyses: participants with subthreshold or severe psychological distress at baseline, as well as adherent participants, demonstrated a significant increase in specific domains of physical activity. These findings suggest that the app may induce behavioral change in specific subgroups. However, these results should be interpreted with caution, given the large SEs and the fact that these analyses were not prespecified. Further studies are required to elucidate the mechanisms by which these mHealth interventions reduce psychological distress.

In the intervention group, objectively recorded data indicated a small increase in the duration of daily physical activity during the intervention period. However, this finding should also be interpreted with caution due to the measurement imbalance between groups. In addition, the data derived from smartphone-based platforms may vary by operating systems and devices. These factors may have introduced measurement variability. Discrepancies between subjective and objective measures of physical activity were observed in our previous feasibility study [23]. Self-reported physical activity measured using the GPAQ shows only a moderate correlation with accelerometer-based measurements, excludes activities lasting less than 10 minutes, and is often overreported because of social desirability bias [35,50,51]. Conversely, physical activity assessed using stand-alone smartphones can capture short or incidental activities that may not be reported in the GPAQ. However, such measures may miss activity bouts when users do not carry their smartphones. Therefore, these discrepancies may reflect differences in measurement methods, indicating that ASHARE might have primarily influenced short-term or incidental physical activity. Future studies should incorporate comprehensive and comparable assessments of physical activity in both groups.

Interestingly, the subgroup analysis demonstrated a stronger, statistically significant effect among participants with minimal or no psychological distress at baseline (K6 score <5). As physical activity interventions are generally more effective among individuals with elevated symptom levels, this was a distinctive finding in the present trial. Taken together with the observation that increases in physical activity were not clearly demonstrated, this result suggests that the mechanism through which ASHARE reduces psychological distress may not solely involve increased physical activity. One possible explanation is that continuous monitoring may help generally healthy workers maintain stable, healthy physical activity patterns, thereby contributing to the stabilization of psychological distress levels over time. However, because improvements in physical activity were not clearly demonstrated in this study, this interpretation remains speculative. Further investigation is warranted in future studies.

### Limitations

This trial has several limitations that should be considered when interpreting the findings. Although the follow-up rates at 3 and 6 months exceeded 85% in both groups, attrition bias might have influenced the results if participants with worsening psychological distress were more likely to withdraw from the trial. More than 20% of the participants in the intervention group and more than half of those in the control group did not receive the allocated program even though they received a download link via email. These results might reflect low intervention fidelity and may have introduced bias due to deviations from the intended intervention. Given that the per-protocol analysis demonstrated a greater decrease in psychological distress and an increase in leisure-time physical activity, the true effects and potential mechanisms of the app might differ from those observed in the main analysis. The present findings should be interpreted primarily as an evaluation of effectiveness under routine conditions rather than efficacy under optimal adherence. In addition, detailed usage metrics for app users, such as time spent per session, were not available; therefore, actual daily interactions with the app could not be fully quantified. As allocation blinding was not feasible, information bias might have occurred in the assessment of self-reported outcomes. Because participants were aware of their group assignment, the observed short-term reduction in psychological distress may partly reflect expectancy or reporting effects. Importantly, there was a substantial imbalance in contact time between the intervention and control groups. This may have influenced the observed effects on the outcomes, which may not be attributable solely to the intervention itself but also to differences in contact with the research team. Additional contact through reminder emails and optional Zoom sessions may have enhanced participants’ engagement and expectations regarding intervention effectiveness. Future studies should employ balanced control conditions to differentiate the specific effects of the intervention from those associated with differential participant contact. Generalizability may be limited to workers who were interested in the research topic and who had their own compatible smartphones, and the findings may not extend to broader workplace settings or to people less familiar with digital tools.

### Conclusions

The stand-alone smartphone-based app, ASHARE, modestly reduced psychological distress among workers. However, the benefit may be short-lived and not durable and may have limited clinical and practical significance. In addition, it remains unclear whether the app reduces psychological distress through increased physical activity. Although the findings of this trial suggest that mHealth interventions that enable workers to monitor their physical activity and mental health may contribute to preventing mental health problems in workplace settings, booster engagement strategies are necessary to sustain benefits over time. Subgroup analyses suggest that the app may be more effective in generally healthy populations. Future studies should examine the effectiveness of such interventions on physical activity and evaluate their long-term effectiveness.

## Supplementary material

10.2196/96072 Multimedia Appendix 1 Implementation outcomes and the potential harm of ASHARE evaluated at the 3-month follow-up.

10.2196/96072 Multimedia Appendix 2 Effects of the intervention program on the amount of physical activity (N=793).

10.2196/96072 Multimedia Appendix 3 Physical activity duration based on the digitally recorded data from the app (n=307).

10.2196/96072 Multimedia Appendix 4 Subgroup analysis: effects of the intervention program on the amount of physical activity by levels of psychological distress.

10.2196/96072 Multimedia Appendix 5 The per-protocol analysis among adherent participants (n=279).

10.2196/96072 Checklist 1 CONSORT checklist.

## Abbreviations

CONSORT: Consolidated Standards of Reporting Trials
GPAQ: Global Physical Activity Questionnaire
iOSDMH: Implementation Outcome Scale of Digital Mental Health
K6: 6-item Kessler Psychological Distress Scale
M-ORION: Multifaceted Organizational Interventions
MET: metabolic equivalent of task
mHealth: mobile health
OR: odds ratio
RCT: randomized controlled trial
WHO: World Health Organization
