# Supplementary material for: An mHealth Intervention Promoting Physical Activity to Reduce Psychological Distress Among Workers: Randomized Controlled Trial
Source: J Med Internet Res. 2026 Aug 14;28:e96072. doi: 10.2196/96072 (PMC13475939; doi:10.2196/96072)
Supplement: Supplementary file 1 [file jmir-v28-e96072-s001.docx]

**Appendix 2. Implementation outcomes and potential harm of ASHARE were evaluated at the three-month follow-up.**

**Supplement Table 1-1. Scores of iOSDMH.**

|  | The present RCT  (n = 347) | The previous non-RCT^a^  (n = 61) | The previous feasibility trial^b^  (n = 23) | Other mHealth studies^c^ | | | | | |
| --- | --- | --- | --- | --- | --- | --- | --- | --- | --- |
|  |  |  |  | Study 1  (n = 934) | Study 2  (n= 142) | Study 3  (n = 474) | Study 4  (n = 235) | Study 5  (n = 131) | Study 6  (n = 29) |
|  | Mean (SD) | Mean (SD) | Mean (SD) | Mean (SD) | Mean (SD) | Mean (SD) | Mean (SD) | Mean (SD) | Mean (SD) |
| Overall score  (14 items; 14–56) | 39.74 (7.4) | 36.21 (7.2) | 36.65 (5.1) | 41.3 (5.7) | 39.7 (8.7) | 41.3 (6.9) | 36.3 (6.3) | 46.3 (6.5) | 38.8 (7.4) |
| Acceptability  (3 items; 3–12) | 7.76 (1.9) | 7.05 (1.9) | 6.87 (1.5) | 8.0 (1.5) | 8.4 (2.2) | 8.7 (1.7) | 7.6 (1.7) | 9.6 (1.8) | 8.0 (2.1) |
| Appropriateness  (4 items; 4–16) | 10.87 (2.6) | 9.87 (2.4) | 9.96 (2.3) | 11.9 (2.0) | 11.5 (3.0) | 11.4 (2.3) | 10.1 (2.3) | 13.0 (2.4) | 12.3 (2.4) |
| Feasibility  (6 items; 6–24) | 18.48 (3.4) | 17.00 (3.5) | 17.74 (2.8) | 18.5 (2.8) | 17.0 (3.6) | 18.3 (3.2) | 16.0 (2.7) | 20.3 (3.0) | 15.8 (3.6) |
| Satisfaction  (1 item; 1–4) | 2.63 (0.7) | 2.30 (0.8) | 2.09 (0.7) | 3.0 (0.6) | 2.9 (0.9) | 3.0 (0.7) | 2.6 (0.7) | 3.4 (0.7) | 2.8 (0.9) |
| Harm  (5 items; 5–20) | 7.36 (3.0) | 7.10 (2.8) | 6.43 (1.6) | 7.4 (2.7) | 9.4 (2.7) | 9.0 (3.3) | 9.3 (3.0) | 8.2 (3.1) | 9.2 (3.2) |

Note. RCT: randomized controlled trial. a: scores were obtained in our previous non-RCT (Watanabe et al., JMIR Mhealth Uhealth. 2025;13:e70473. doi: 10.2196/70473). b: scores were obtained in our previous feasibility trial (Watanabe et al., JMIR Form Res. 2023;7:e51334. doi: 10.2196/51334). c: scores were reported by the previous systematic review (Obikane et al., Int J Environ Res Public Health. 2022;19(23):15792. doi: 10.3390/ijerph192315792).

**Supplement Table 1-2. Responses for items of the harm subscale (n = 347)**

| Harms | Disagree | Relatively disagree | Relatively agree | Agree | Harms  (Relatively agree and Agree) |
| --- | --- | --- | --- | --- | --- |
|  | n (%) | n (%) | n (%) | n (%) | % |
| Using this program causes physical symptoms  (e.g., Tired eyes, headache, stiffness shoulders)  (Physical symptoms) | 246 (70.9) | 70 (20.2) | 26 (7.5) | 5 (1.4) | 8.9 |
| Using this program causes mental symptom  (e.g., depression, insomnia)  (Mental symptoms) | 250 (72.0) | 70 (20.2) | 25 (7.2) | 2 (0.6) | 7.8 |
| Using this program sometimes brings us a smart phone induced dangerous experience regarding safety  (e.g., collide with people while walking and looking at the smart phone)  (Induced dangerous experience regarding safety) | 261 (75.2) | 66 (19.0) | 18 (5.2) | 2 (0.6) | 5.8 |
| I have a concern that the use of this program consumes my time for other activities  (e.g., time for leisure, family affairs, sleep, education)  (Time-consuming) | 220 (63.4) | 79 (22.8) | 35 (10.1) | 13 (3.7) | 13.8 |
| Using this program makes me face the excessive pressure on learning this program regularly  (Excessive pressure on learning regularly) | 175 (50.4) | 103 (29.7) | 51 (14.7) | 18 (5.2) | 19.9 |
