# Supplementary material for: An mHealth Intervention Promoting Physical Activity to Reduce Psychological Distress Among Workers: Randomized Controlled Trial
Source: J Med Internet Res. 2026 Aug 14;28:e96072. doi: 10.2196/96072 (PMC13475939; doi:10.2196/96072)
Supplement: Supplementary file 2 [file jmir-v28-e96072-s002.docx]

**Appendix 3. Effects of the intervention program on the amount of physical activity (n = 793).**

|  | **Intervention**  **(n=397)** | **Control**  **(n=396)** | **Differences in change scores of outcomes** | | | |
| --- | --- | --- | --- | --- | --- | --- |
| **Amount of physical activity (GPAQ, METs-Min)** | **Estimated mean**  **(95% CI)** | | **Estimated mean**  **difference**  **(95% CI)** | **Pooled**  **SD** | **Effect size**  **(Cohen’s d)**  **(95% CI)** | **p-value** |
| Total activity |  |  |  |  |  |  |
| Baseline | 2416.29  (2088.79 to 2743.78) | 2690.13  (2296.54 to 3083.72) | – | – | – | – |
| 3-month follow-up | 2572.99  (2172.67 to 2973.31) | 2638.81  (2260.40 to 3107.21) | 208.03  (-392.31 to 808.37) | 4026.06 | 0.05  (-0.10 to 0.20) | 0.497 |
| 6-month follow-up | 2226.03  (1866.35 to 2585.70) | 2172.77  (1771.25 to 2574.28) | 327.10  (-265.94 to 920.15) | 3943.60 | 0.08  (-0.07 to 0.23) | 0.280 |
| Occupational activity |  |  |  |  |  |  |
| Baseline | 803.27  (596.15 to 1010.38) | 1002.76  (765.67 to 1239.84) | – | – | – | – |
| 3-month follow-up | 842.77  (594.80 to 1090.74) | 1055.33  (818.27 to 1292.39) | -13.07  (-384.15 to 358.01) | 2413.16 | -0.01  (-0.16 to 0.15) | 0.945 |
| 6-month follow-up | 767.27  (500.18 to 1034.37) | 770.78  (442.60 to 1098.95) | 195.99  (-238.85 to 630.83) | 2829.31 | 0.07  (-0.08 to 0.22) | 0.377 |
| Transport activity |  |  |  |  |  |  |
| Baseline | 927.93  (753.00 to 1102.87) | 1001.67  (769.70 to 1233.63) | – | – | – | – |
| 3-month follow-up | 1005.28  (793.29 to 1217.27) | 893.32  (683.53 to 1103.12) | 185.69  (-210.61 to 582.00) | 2760.49 | 0.07  (-0.08 to 0.21) | 0.358 |
| 6-month follow-up | 846.52  (709.02 to 984.03) | 824.36  (687.11 to 961.61) | 95.90  (-227.69 to 419.49) | 2297.82 | 0.04  (-0.10 to 0.18) | 0.561 |
| Leisure-time activity |  |  |  |  |  |  |
| Baseline | 685.09  (566.63 to 803.55) | 685.71  (554.18 to 817.23) | – | – | – | – |
| 3-month follow-up | 724.94  (577.77 to 872.12) | 690.15  (558.12 to 822.19) | 35.41  (-154.92 to 225.73) | 1246.87 | 0.03  (-0.12 to 0.18) | 0.715 |
| 6-month follow-up | 612.23  (484.86 to 739.60) | 577.63  (465.96 to 689.31) | 35.21  (-135.43 to 205.85) | 1111.93 | 0.03  (-0.12 to 0.19) | 0.686 |
