# Supplementary material for: An mHealth Intervention Promoting Physical Activity to Reduce Psychological Distress Among Workers: Randomized Controlled Trial
Source: J Med Internet Res. 2026 Aug 14;28:e96072. doi: 10.2196/96072 (PMC13475939; doi:10.2196/96072)
Supplement: Supplementary file 4 [file jmir-v28-e96072-s004.docx]

**Appendix 5. Subgroup analysis: Effects of the intervention program on the amount of physical activity by levels of psychological distress.**

|  | **No or minimal (K6 <5)** | | | **Subthreshold or severe (K6 ≥5)** | | |
| --- | --- | --- | --- | --- | --- | --- |
|  | **Differences in change scores of outcomes** | | | **Differences in change scores of outcomes** | | |
| **Amount of physical activity (GPAQ, METs-Min)** | **Estimated mean**  **difference**  **(95% CI)** | **Effect size**  **(Cohen’s d)**  **(95% CI)** | **p-value** | **Estimated mean**  **difference**  **(95% CI)** | **Effect size**  **(Cohen’s d)**  **(95% CI)** | **p-value** |
| Total activity (METs-Min) |  |  |  |  |  |  |
| 3-month follow-up | -399.86  (-1161.99 to 362.28) | -0.10  (-0.29 to 0.09) | 0.304 | **1017.21**  **(52.20 to 1982.21)** | **0.25**  **(0.01 to 0.49)** | **0.039** |
| 6-month follow-up | 90.46  (-657.39 to 838.31) | 0.02  (-0.17 to 0.21) | 0.813 | 641.50  (-284.11 to 1567.10) | 0.16  (-0.07 to 0.40) | 0.174 |
| Occupational activity |  |  |  |  |  |  |
| 3-month follow-up | -151.74  (-591.05 to 287.57) | -0.06  (-0.24 to 0.12) | 0.498 | 171.53  (-473.83 to 816.90) | 0.07  (-0.20 to 0.34) | 0.602 |
| 6-month follow-up | 228.03  (-327.38 to 783.45) | 0.08  (-0.12 to 0.28) | 0.421 | 152.46  (-519.96 to 824.89) | 0.05  (-0.18 to 0.29) | 0.657 |
| Transport activity |  |  |  |  |  |  |
| 3-month follow-up | -125.90  (-706.99 to 455.18) | -0.05  (-0.26 to 0.16) | 0.671 | **600.54**  **(83.18 to 1117.90)** | **0.22**  **(0.03 to 0.40)** | **0.023** |
| 6-month follow-up | -86.63334  (-543.26 to 369.99) | -0.04  (-0.24 to 0.16) | 0.710 | 339.03  (-98.23 to 776.30) | 0.15  (-0.04 to 0.34) | 0.129 |
| Leisure-time activity |  |  |  |  |  |  |
| 3-month follow-up | -122.21  (-368.23 to 123.81) | -0.10  (-0.30 to 0.10) | 0.330 | 245.13  (-50.93 to 541.18) | 0.20  (-0.04 to 0.43) | 0.105 |
| 6-month follow-up | -50.94  (-260.28 to 158.40) | -0.05  (-0.23 to 0.14) | 0.633 | 150.00  (-135.35 to 435.35) | 0.13  (-0.12 to 0.39) | 0.303 |
