# Supplementary material for: An mHealth Intervention Promoting Physical Activity to Reduce Psychological Distress Among Workers: Randomized Controlled Trial
Source: J Med Internet Res. 2026 Aug 14;28:e96072. doi: 10.2196/96072 (PMC13475939; doi:10.2196/96072)
Supplement: Supplementary file 5 [file jmir-v28-e96072-s005.docx]

**Appendix 6. The per-protocol analysis among adherent participants (n = 279).**

**Supplement Table 6-1. Effects of the intervention program on psychological distress**

|  | Intervention  (n = 120) | Control  (n = 159) | Differences in change scores of outcomes | | | |
| --- | --- | --- | --- | --- | --- | --- |
| Psychological distress (K6) | Estimated mean  (95% CI) | | Estimated mean  difference  (95% CI) | Pooled  SD | Effect size  (Cohen’s d)  (95% CI) | p-value |
| Baseline | 4.89  (4.38 to 5.41) | 4.66  (4.20 to 5.12) | – | – | – | – |
| 3-month follow-up | 4.48  (3.80 to 5.17) | 5.17  (4.51 to 5.82) | **-0.92**  **(-1.65 to -0.18)** | **3.31** | **-0.28**  **(-0.59 to -0.06)** | **0.014** |
| 6-month follow-up | 4.57  (3.87 to 5.27) | 4.83  (4.20 to 5.47) | -0.50  (-1.28 to 0.28) | 3.30 | -0.15  (-0.39 to 0.09) | 0.209 |

Note. CI: confidence interval. SD: standard deviation. Global Physical Activity Questionnaire. The estimated mean scores were adjusted according to the level of psychological distress at baseline (0=no or minimal distress; 1=subthreshold to severe distress).

**Supplement Table 6-2. Effects of the intervention program on the level of physical activity**

|  |  | Intervention  (n = 120) | Control  (n = 159) | Differences in change levels | | |
| --- | --- | --- | --- | --- | --- | --- |
| Level of physical activity  (GPAQ) | | Estimated probability (%)  (95% CI) | | Estimated difference  (95% CI) | Cumulative OR  (95% CI) | p-value |
| Baseline | High | 10.7  (4.8 to 16.6) | 9.9  (5.0 to 14.7) | – | – | – |
|  | Moderate | 61.6  (53.9 to 69.3) | 60.6  (53.1 to 68.1) |  |  |  |
|  | Low | 27.7  (15.9 to 39.5) | 29.5  (19.0 to 40.1) |  |  |  |
| 3-month follow-up | High | 10.5  (4.8 to 16.6) | 8.5  (4.2 to 12.9) | 0.13  (-0.61 to 0.88) | 1.14  (0.54 to 2.41) | 0.723 |
|  | Moderate | 61.3  (53.5 to 69.2) | 58.5  (50.2 to 66.9) |  |  |  |
|  | Low | 28.2  (16.2 to 40.1) | 32.9  (21.7 to 44.2) |  |  |  |
| 6-month follow-up | High | 8.8  (3.8 to 13.9) | 6.6  (3.1 to 10.1) | 0.23  (-0.52 to 0.97) | 1.25  (0.60 to 2.64) | 0.551 |
|  | Moderate | 59.0  (49.9 to 68.1) | 54.0  (44.4 to 63.7) |  |  |  |
|  | Low | 32.2  (19.4 to 44.9) | 39.4  (27.2 to 51.5) |  |  |  |

Note. GPAQ: Global Physical Activity Questionnaire. CI: confidence interval. OR: odds ratio. High: ≥3 days of vigorous intensity activity for ≥1,500 MET-min/week or ≥7 days of any combination of walking or moderate to vigorous intensity physical activity for 3,000 MET-min/week; moderate: ≥3 days of vigorous intensity activity for ≥20 min/day or ≥5 days of moderate intensity activity or walking for ≥30 min/day or ≥5 days of any combination of walking or moderate to vigorous intensity physical activity for ≥600 MET-min/week; low: not meeting the criteria for high or moderate.

**Supplement Table 6-3. Effects of the intervention program on the amount of physical activity**

|  | **Intervention**  **(n=120)** | **Control**  **(n=159)** | **Differences in change scores of outcomes** | | | |
| --- | --- | --- | --- | --- | --- | --- |
| **Amount of physical activity (GPAQ, METs-Min)** | **Estimated mean**  **(95% CI)** | | **Estimated mean**  **difference**  **(95% CI)** | **Pooled**  **SD** | **Effect size**  **(Cohen’s d)**  **(95% CI)** | **p-value** |
| Total activity |  |  |  |  |  |  |
| Baseline | 2401.37  (1776.85 to 3025.89) | 2274.72  (1771.12 to 2278.31) | – | – | – | – |
| 3-month follow-up | 2038.37  (1568.68 to 2508.06) | 2216.33  (1706.23 to 2726.43) | -304.61  (-1136.47 to 527.25) | 4026.06 | -0.08  (-0.28 to 0.13) | 0.473 |
| 6-month follow-up | 2206.54  (1534.12 to 2878.96) | 1852.48  (1261.47 to 2443.49) | 227.41  (-664.11 to 1118.92) | 3943.60 | 0.06  (-0.17 to 0.28) | 0.617 |
| Occupational activity |  |  |  |  |  |  |
| Baseline | 955.06  (507.16 to 1402.97) | 679.55  (449.69 to 909.41) | – | – | – | – |
| 3-month follow-up | 600.53  (308.00 to 893.06) | 834.14  (554.29 to 1113.99) | -509.12  (-1022.86 to 4.62) | 2413.16 | -0.21  (-0.42 to 0.00) | 0.052 |
| 6-month follow-up | 926.73  (316.98 to 1536.48) | 755.63  (183.53 to 1327.73) | -104.41  (-802.78 to 593.97) | 2829.31 | -0.04  (-0.28 to 0.21) | 0.770 |
| Transport activity |  |  |  |  |  |  |
| Baseline | 901.26  (637.29 to 1165.25) | 977.28  (631.27 to 1323.30) | – | – | – | – |
| 3-month follow-up | 713.37  (557.93 to 868.81) | 874.26  (516.17 to 1232.36) | -84.88  (-630.12 to 460.35) | 2760.49 | -0.03  (-0.23 to 0.17) | 0.760 |
| 6-month follow-up | 772.27  (595.42 to 949.12) | 678.54  (543.56 to 813.52) | 169.74  (-287.95 to 627.43) | 2297.82 | 0.07  (-0.13 to 0.27) | 0.467 |
| Leisure-time activity |  |  |  |  |  |  |
| Baseline | 545.94  (367.50 to 722.57) | 617.88  (385.47 to 850.30) | – | – | – | – |
| 3-month follow-up | 724.47  (455.79 to 993.15) | 507.92  (348.47 to 667.37) | **289.40**  **(17.20 to 561.60)** | **1246.87** | **0.23**  **(0.01 to 0.45)** | **0.037** |
| 6-month follow-up | 507.54  (338.52 to 676.55) | 418.31  (285.31 to 551.31) | 162.07  (-91.47 to 415.61) | 1111.93 | 0.15  (-0.08 to 0.37) | 0.210 |
