# Supplementary material for: An mHealth Intervention Promoting Physical Activity to Reduce Psychological Distress Among Workers: Randomized Controlled Trial
Source: J Med Internet Res. 2026 Aug 14;28:e96072. doi: 10.2196/96072 (PMC13475939; doi:10.2196/96072)
Supplement: Supplementary file 7 [file jmir-v28-e96072-s003.docx]

**Appendix 4. Physical activity duration based on the digitally recorded data from the app (n = 307).**
